# The disposition effect in a scopic regime: Data from a laboratory experiment^☆^

**DOI:** 10.1016/j.dib.2020.105680

**Published:** 2020-05-12

**Authors:** Minh-Lý Liêu, Matthias Pelster

**Affiliations:** Paderborn University, Warburger Str. 100, Paderborn 33098, Germany

**Keywords:** Social interactions, Social comparisons, Scopic regime, Investor behavior, Online trading

## Abstract

This article presents a new dataset on the disposition effect in a scopic regime, collected in a laboratory experiment reported in “Framing and the disposition effect in a scopic regime” [6]. 81 subjects were recruited and asked to participate in an incentivized stock trading game insprired by Weber and Camerer [7], which was computerized, programmed using oTree [1]. Subjects were able to monitor the trading performance of randomly selected peers in comparison to their own performance. Two sets of rankings were employed to display the comparisons. The data allow to test theories on the impact of social comparisons on investors’ decision making.

Specifications table

**Table:** 

Subject area	Finance
Specific subject area	Behavioral Finance, Social Finance, Experimental Finance, Trading Behavior, Investor Behavior
Type of data	Subjects’ decisions from incentivized laboratory experiments
How data were acquired	Collecting participant choices in an economic
	laboratory experiment using OTree [1]
Data format	Raw (csv-file or data.table in Rdata-file)
	Data is organized in a panel structure
	Period-invariant data is assigned the last trading period
Parameters for data collection	Performance ranking (percentage of profitable trades
	vs. raw performance (portfolio value))
Description of data collection	Data were collected in a laboratory experiment and contain individual choices in a scopic regime
Data Source Location	Paderborn University, Germany
Data accessibility	DOI: 10.17632/jfg8s32xdm (https://data.mendeley.com/datasets/jfg8s32xdm).
Related research article	Liêu, M.-L. & Pelster, M., Framing and the disposition effect in a scopic regime,
	The Quarterly Review of Economics and Finance, 2020, DOI:10.1016/j.qref.2020.01.008.

## Value of the data

• The data can be used to test theories on the impact of social comparisons on decision making or to set a benchmark for formulating a new theory of decision making in a scopic regime.
• The data can be used to test existing disposition effect theories.
• This data can be combined with other experimental data on the disposition effect to find undiscovered findings.

## 1 Data description

The data is stored in one file, containing the entire raw data collected during the experiment. The data contain observations from an incentivized experiment, conducted in June 2019 at the BaER-Lab at Paderborn University. 81 subjects were recruited through ORSEE [4]. The experiment was programmed using oTree [1]. The subjects were incentivized as we paid cash proportional to the profit earned in the experiment.

Participants completed the multi-period stock trading task inspired by Weber and Camerer [7]. The data contain every decision of investors across periods. Participants were able to monitor the performance of two randomly selected peers during the experiment and compare their peers’ performance to their own. Two different types of ranking were employed to frame the comparison with peers. The data contain detailed information on the randomly composed peer groups and the type of ranking per treatment condition. The dataset also includes information on the participants’ risk preferences [2], [5] and loss aversion [3] as well as standard demographics such as age and gender.

The data can be found online at https://data.mendeley.com/datasets/jfg8s32xdm. Definitions of all variables in the dataset are given in Table 1.

**Table 1:** Variable definitions.

Variable	Variable Name	Variable Definition
Session identifier	session.code	Primary key for each session
Participant identifier	participant.code	Primary key for each participant
Condition variable	participant_current_app	denotes treatment condition of participant
Peer group	group.id_in_subsession	Randomly selected peer group
Period	subsession.round_number	Current period
Age	player.age	Years
Gender	player.gender	Male for men, Female for women
Major in finance	player.finance	Yes if Finance-related major
Major in business	player.business	Yes if Business-related major
Major in economics	player.economics	Yes if Economics-related major
Experience in trading	player.selfassessment	1 if no experience;... 10 if high experience
Recognize participants	player.recognize_participants	Did participant recognize other participants in session?
Risk preference task [5]	player.holt_laury“j”	Participant’s decision on “j”th lottery (j = 1 to 10)
Risk preference task [2]	player.lottery	Participant’s selected lottery
Loss preference task [3]	player.coin_“j”	Participant’s decision on “j”th coinflip (j = 1 to 10)
Initial stock holdings “i”	player.quantity_pre_“i”	Quantity of stock “i” at the beginning of period; i = A,B,C,D,E,F
Stock sales “i”	player.quantity_sell_“i”	Quantity of sold stocks “i”
Stock purchases “i”	player.quantity_buy_“i”	Quantity of bought stocks “i”
Final stock holdings “i”	player.quantity_“i”	Quantity of stock “i” at the end of period
Price “i”	player.price_“i”	Price of stock “i”
Portfolio value	player.result_final	Participants’ portfolio value
Guess task	player.total_count_guess	Sum of correct stock type guesses

## 2 Experimental design, materials and methods

A total of six sessions of the experiment were conducted. Each session was conducted in two parts. Part I included the trading task, part II included the additional collection of participants’ demographics, risk preferences, and loss aversion.

In part I, a total of 14 periods of the trading task based on Weber and Camerer [7] were run. Subjects were endowed with fictional wealth of 10,000 Talers. With their endowment, subjects were able to trade six different stocks with uncertain return characteristics over 14 periods. Stock prices followed distinct random processes and were not influenced by investment decisions of participants. The six stocks represented different stock types with different chances to increase their prices. The stock with the best expected performance had a 65% chance of a positive price change; the second best stock had a 55% chance of a positive price change; two neutral stocks had even probability of a positive or negative price change; the fifth stock had a 55% chance of a decrease in stock price; the stock with the worst expected performance had a 65% chance of a decrease in stock price. Participants were informed about the existence of different stocks and their characteristics, but did not get any information which stock possesses which characteristics. After the direction of stock price changes had been determined, as second stochastic process determined the magnitude of the price change. Both stochastic processes were independent. Price increases were measured in terms of absolute values and could be either 1, 3, or 5 Talers. Participants were be informed about the two-stage pricing process before the experiment. Participants received information on the stock prices of four periods (-3, -2, -1, 0) before period one started. To evaluate whether subjects had a good understanding of the stock types, they had to guess the stock types after periods seven and fourteen.

After rounds five, ten and fourteen, participants were shown rankings with their own performance and the performance of two randomly selected peers. All three rankings were shown with respect to the same peers. Importantly, rankings did not have any implications for participants’ payoffs and this fact was pointed out to participants. In treatment condition spt1, participants were ranked based on their percentage of profitable trades. The fraction of profitable trades denoted the percentage of trades that were closed at a price, which was higher than the entry price. In addition, the rankings information displayed the raw performance (current portfolio value) of participants. In treatment condition spt2, participants were ranked based on their raw performance (current portfolio value). No information about the percentage of profitable trades were given in this treatment condition.

In part II of the experiment, information on participants’ gender, age, university major, stock market experience, and major were collected. Additionally, participants had to complete the risk elicitation tasks of Eckel and Grossman [2] and Holt and Laury [5] as well as the loss aversion elicitation task of Gächter et al. [3]. The risk elicitation tasks and the loss aversion elicitation task were incentivized.

## Appendix A Supplementary materials

Supplementary Data S1 Supplementary Raw Research Data. This is open data under the CC BY license http://creativecommons.org/licenses/by/4.0/ Supplementary Data S1
